# Concordance of Sleep and Pain Outcomes of Diverse Interventions: An Umbrella Review

**DOI:** 10.1371/journal.pone.0040891

**Published:** 2012-07-17

**Authors:** Anthony G. Doufas, Orestis A. Panagiotou, John P. A. Ioannidis

**Affiliations:** 1 Department of Anesthesia, Stanford University School of Medicine, Stanford, California, United States of America; 2 Outcomes Research Consortium, Cleveland, Ohio, United States of America; 3 Department of Hygiene and Epidemiology, University of Ioannina School of Medicine, Ioannina, Greece; 4 Stanford Prevention Research Center, Department of Medicine, Stanford University School of Medicine, Stanford, California, United States of America; 5 Department of Health Research and Policy, Stanford University School of Medicine, Stanford, California, United States of America; 6 Department of Statistics, Stanford University School of Humanities and Sciences, Stanford, California, United States of America; Imperial College London, United Kingdom

## Abstract

**Background/Objective:**

Pain influences sleep and vice versa. We performed an umbrella review of meta-analyses on treatments for diverse conditions in order to examine whether diverse medical treatments for different conditions have similar or divergent effects on pain and sleep.

**Methods:**

We searched published systematic reviews with meta-analyses in the Cochrane Database of Systematic Reviews until October 20, 2011. We identified randomized trials (or meta-analyses thereof, when >1 trial was available) where both pain and sleep outcomes were examined. Pain outcomes were categorized as headache, musculoskeletal, abdominal, pelvic, generic or other pain. Sleep outcomes included insomnia, sleep disruption, and sleep disturbance. We estimated odds ratios for all outcomes and evaluated the concordance in the direction of effects between sleep and various types of pain and the correlation of treatment effects between sleep and pain outcomes.

**Results:**

151 comparisons with 385 different trials met our eligibility criteria. 96 comparisons had concordant direction of effects between each pain outcome and sleep, while in 55 the effect estimates were in opposite directions (P<0.0001). In the 20 comparisons with largest amount of evidence, the experimental drug always had worse sleep outcomes and tended to have worse pain outcomes in 17/20 cases. For headache and musculoskeletal pain, 69 comparisons showed concordant direction of effects with sleep outcomes and 36 showed discordant direction (P<0.0001). For the other 4 pain types there were overall 27 vs. 19 pairs with concordant vs. discordant direction of effects (P = 0.095). There was a weak correlation of the treatment effect sizes for sleep vs. headache/musculoskeletal pain (r = 0.17, P = 0.092).

**Conclusions:**

Medical interventions tend to have effects in the same direction for pain and sleep outcomes, but exceptions occur. Concordance is primarily seen for sleep and headache or musculoskeletal pain where many drugs may both disturb sleep and cause pain.

## Introduction

Sleep is a critical process of life that is often under threat by acute or chronic pain. Dysfunctional sleep and chronic pain are two major, yet unmet, public health challenges with an enormous societal cost [1]–[4]. Over a third of the US population is affected by a chronic pain condition, while a fifth suffer from sleep disorders, which degrade daily function and may lead to metabolic and cardiovascular morbidity [2], [4]. Twenty percent of adults report that pain disturbs their sleep a few nights a week or more [5], while back pain, headaches, and muscle aches are the most common types of pain experienced at night [6]. Approximately 10% of patients seen in primary care report major insomnia [7] and sleep disturbances exist in 50–89% [8]–[10] or more [11], [12] patients with chronic pain. Reciprocally, patients with primary sleep disorders [13]–[15] are far more likely to suffer from chronic pain diseases like fibromyalgia, rheumatoid arthritis, temporomandibular joint disorder, or headaches [16]. Both experimental [15]–[19] and preliminary clinical [20]–[22] evidence support a complicated, circular model of influence between the functions of sleep and pain [17], [23]. Patients with impaired sleep may possess genetic or physiological traits that facilitate the development or exacerbate certain types of chronic pain behavior with or without the occurrence of an opportune acute injury [20]–[22], [24]–[26]. Ultimately, unremitting pain may further disrupt sleep perpetuating a vicious cycle.

Many treatments given for diverse conditions may cause sleep or pain problems or may aim at improving pain and/or sleep as primary or secondary outcomes. It is unknown whether the effects of different treatments in diverse settings on sleep and pain are concordant, and whether situations exist where responses in these two outcomes are different or even in the opposite direction. It would be useful to dissect the concordance between these outcomes for different types of pain. To explore these issues, we performed an umbrella review that encompassed a large number of systematic reviews with meta-analysis of clinical trials on topics where data on both pain- and sleep-related outcomes were available.

## Methods

### Eligibility Criteria

We considered Cochrane systematic reviews including separate data on binary pain- and sleep-related outcomes during follow-up for the comparison of the same experimental treatment against the same comparator (placebo, no treatment, or other treatment). We included comparisons regardless of the number of trials with data for each outcome. We also accepted comparisons for any disease or condition. We excluded protocols; reviews where the assessed outcomes did not include at least one pain-related and at least one sleep-related outcome; and comparisons where it was unclear which one was the experimental intervention among those compared (comparisons of different doses/formulations of the same intervention, different agents in the same class, or different interventions where it was unclear which one is the standard of care).

Whenever assessments for either pain or sleep were performed at several different time points we retained the data for the time points with the largest number of studies. We accepted reviews regardless of whether the pain- or sleep-related assessments pertained to the evaluation of outcome status or change (improvement or deterioration).

#### Search strategy

We searched the Cochrane Library database (last search performed October 20, 2011) using the terms “sleep” and “insomnia” for sleep, and “pain”, “headache”, “migraine”, “myalgia”, “arthralgia”, “backache”, and “ache” for pain. Whenever reviews contained >1 eligible comparison, these were considered separately.

### Sleep and Pain Outcomes

Eligible sleep-related assessments were considered together and included outcomes classified as insomnia, sleep disruption and sleep disturbance. We excluded outcomes named somnolence, drowsiness, sedation, abnormal dreams, and hypersomnia. Pain-related outcomes were categorized into 6 groups: headache, musculoskeletal, abdominal, pelvic, generic, and other pain. A detailed description of the specific assessments contained under these categories appears in Table S1 and Table S2. When a pain outcome was listed as generic, we examined carefully the context to see whether it belonged in any of the 5 specific categories.

### Extracted Data

Two authors independently documented eligible comparisons and discrepancies were settled with discussion. In each eligible comparison we recorded the trials with data on the eligible pain and sleep outcomes, noting any overlap and capturing the year of publication, first author, and outcome definitions. Finally, we extracted the 2×2 tables for outcome status or change from baseline. When both were available, we preferred change from baseline.

### Standardization of Outcome Metrics

We estimated the odds ratio (OR) for all outcomes. We consistently coined the comparisons to reflect the contrast of the experimental treatment versus control and consistently to reflect pain or sleep problems or worsening in pain or sleep problems. This means that when the data reflected the number of patients who had no pain (e.g., 10/36), we took the complementary counts (i.e., 26/36); and whenever the data reflected the number of patients with improvement in pain, we took again the complementary counts. The same rule was applied to sleep outcomes. Therefore, whenever the experimental treatment is better, the OR is <1.00.

### Analyses

Multiple trials on the same comparison were synthesized per fixed-effect models [27]. Sensitivity analyses used random-effects [27] and the results are highly similar (not shown in detail), because in most topics there was either only one trial or a few trials and no demonstrable between-trial heterogeneity. We evaluated whether the point estimates for pain and sleep outcomes were in the same or opposite direction and whether they were nominally statistically significant in the same or opposite direction. Direction of effect pertains to the point estimates of the treatment effects and these have large uncertainty when there are limited data. Therefore, we also examined how often the 95% confidence intervals (CIs) excluded a null-effect for both outcomes, and, if so, whether these effects were in the same or opposite direction; how many of the 95% CIs of pain and sleep outcomes did not overlap; and how many of the 95% CIs of pain and sleep outcomes differed beyond chance (*p*<0.05).

To assess whether the magnitude of the treatment effect size correlated for pain and sleep outcomes, we estimated the Pearson correlation coefficients weighted by the inverse of the sum of the variances (squares of the standard errors) of the effects for pain and sleep. This means that observations with limited amount of evidence on either sleep or pain carried less weight in the calculations. We also estimated correlation coefficients without weighting.

There is clinical [14], [16], [17], [21], [28]–[32] and experimental [15], evidence that sleep tends to co-exist more with headache and musculoskeletal pain in particular. Therefore, we hypothesized that headache and musculoskeletal pain may have stronger concordance with sleep outcomes than other types of pain. These two types of pain were considered together and the other types of pain were considered as a separate group. We also hypothesized that the concordance between sleep and pain outcomes may be stronger when these are primary outcomes. Thus, we evaluated separately comparisons where the pain or sleep outcome(s) were considered primary outcomes of the systematic review; and those where neither was among the primary outcomes. We also performed sensitivity analyses limited to trials with data on both sleep and pain outcomes; double-blinded trials; and comparisons of active treatments against no treatment or placebo.

All analyses were performed in STATA 11.2. *p*-values are two tailed.

## Results

### Eligible Data

Sixty-eight Cochrane reviews met the eligibility criteria (Figure S1). These reviews corresponded to 385 different trials pertaining to 151 sleep-pain comparisons (headache, n = 80; musculoskeletal pain, n = 25; abdominal pain, n = 24; pelvic pain, n = 2; generic pain, n = 13; other type of pain, n = 7). The included trials were published between 1961 and 2010 (median 1999; interquartile range, IQR, 1996–2004). The median (IQR) sample size across the 151 comparisons was 432 (127–1999) for pain and 310 (108–968) for sleep outcomes. Table 1 shows the 20 pairs with the largest amount of evidence. They all pertained to drug treatments. Full data on all 151 pairs appear in Table S3.

**Table 1:** Treatment effects for sleep and pain outcomes for the 20 comparisons with largest amount of evidence^a^.

Review	Comparison	OR (95% CI) for sleep	OR (95% CI) for pain	Type of pain
CD000440	Risperidone vs. typical neuroleptic medication for schizophrenia	1.05 (0.83–1.34)	1.06 (0.84–1.36)	Headache
CD001190	Donepezil vs. placebo for dementia due toAlzheimer’s disease	2.16 (1.53–3.04)	1.25 (0.97–1.60)	Headache
CD001396	SSRIs vs. placebo for premenstrual syndrome	1.80 (1.32–2.47)	1.18 (0.92–1.51)	Headache
CD001765	SSRIs vs. placebo for obsessive compulsive disorder	1.96 (1.57–2.45)	1.06 (0.85–1.33)	Headache
CD001867	Naltrexone vs. placebo for alcohol dependence	1.36 (1.04–1.77)	1.01 (0.85–1.19)	Headache
			1.08 (0.76–1.52)	Musculoskeletal pain
			2.30 (1.61–3.27)	Abdominal pain
			1.25 (0.89–1.76)	Generic pain
CD005445	Ribavirin/Interferon vs. interferon for chronic hepatitis C	1.61 (1.37–1.90)	0.84 (0.69–1.04)	Headache
			0.79 (0.59–1.06)	Abdominal pain
			0.95 (0.85–1.06)	Musculoskeletal pain
CD005593	Cholinesterase inhibitor (optimum dose) vs. placebofor Alzheimer’s disease	1.47 (1.10–1.97)	1.47 (1.19–1.82)	Headache
			1.90 (1.40–2.58)	Abdominal pain
CD006103	Varenicline (1.0 mg 2/d) vs. placebo for smoking cessation	1.73 (1.44–2.08)	1.20 (0.99–1.45)	Headache
CD006117	Sertraline vs. TCAs for depression	1.67 (1.21–2.31)	1.31 (0.99–1.74)	Headache
CD006369	Paliperidone - any dose or flexible doses vs. quetiapine(flexible dose all short term) for schizophrenia	1.22 (0.60–2.47)	1.26 (0.65–2.41)	Headache
CD006564	Dopamine agonists vs. placebo/L-dopa in earlyParkinson’s disease	1.28 (1.00–1.62)	1.27 (0.95–1.68)	Headache
CD006622	Aripiprazole vs. placebo for schizophrenia	1.12 (0.84–1.50)	1.16 (0.88–1.54)	Headache
CD007621	Natalizumab + interferon vs. interferon for relapsingremitting multiple sclerosis	1.07 (0.79–1.45)	1.09 (0.86–1.37)	Headache
			1.17 (0.94–1.46)	Musculoskeletal pain

a Amount of evidence is defined by the weight (sum of the inverse variances of the pain and sleep outcome effect sizes), OR>1.00 signifies worse outcome with the experimental versus control treatment.

SSRIs, selective serotonin reuptake inhibitors; OR, odds ratio; CI, confidence interval:, TCA, tricyclic antidepressants.

### Concordance of Direction of Effects

Ninety-six comparisons had concordant direction of effects between each pain outcome and sleep disturbance, and 55 had point estimates of effects in opposite direction (binomial test for concordant vs. discordant direction *p*<0.0001) when all pain outcomes were considered (94 vs. 57 by random-effects calculations).

Among the 20 pairs with the largest amount of evidence, 17 (85%) had effect estimates in the same direction for the two outcomes (Table 1). In all 20 pairs the experimental drug tended to worsen sleep versus the control (statistically significantly so in all but 4 cases). The experimental drug also tended to worsen pain versus the control in 17/20 pairs, and the difference in pain between experimental and control treatment was nominally significant in 3 cases. Effect estimates for sleep and pain outcomes were in the same direction in 14/15 cases where headache or musculoskeletal pain was involved.

### Statistically Significant Effects in the Same and Opposite Direction

We found 6 situations where the pain and sleep disturbance outcomes had nominally statistically significant effects in the same direction. In 5 cases, the experimental interventions worsened both outcomes (sleep and abdominal pain, n = 3 [cholinesterase inhibitor for Alzheimer’s disease [35], naltrexone for alcohol dependence [36], sertraline for depression [37]]; sleep and headache, n = 1 [cholinesterase inhibitor for Alzheimer’s disease [35]]; and sleep and pelvic pain, n = 1 [goserelin versus oral contraceptives for pain associated with endometriosis [38]]), whereas in 1 situation it improved both sleep and musculoskeletal pain (ozzlo pillow for preventing and treating pelvic and back pain in pregnancy [39]). Conversely, there were 3 situations where the pain and sleep disturbance outcomes had nominally statistically significant results in opposite directions. Sertraline versus tricyclics for depression [37] worsened sleep but improved generic pain; GnRH analogs vs placebo worsened sleep, but improved endometriosis-related pelvic pain [40]; and atovaquone-proguanil improved sleep but had worse abdominal pain than amodiaquine when given for uncomplicated malaria [41]. The picture was similar with random effects calculations, except for GnRH analogs versus placebo.

The 95% CIs of the pain and sleep outcomes did not overlap in 12 cases (8%), and the effect estimates differed beyond chance in 23 cases (15%).

### Concordance of Direction of Effects and Correlation of Effect Sizes According to type of Pain

**Figure 1:**
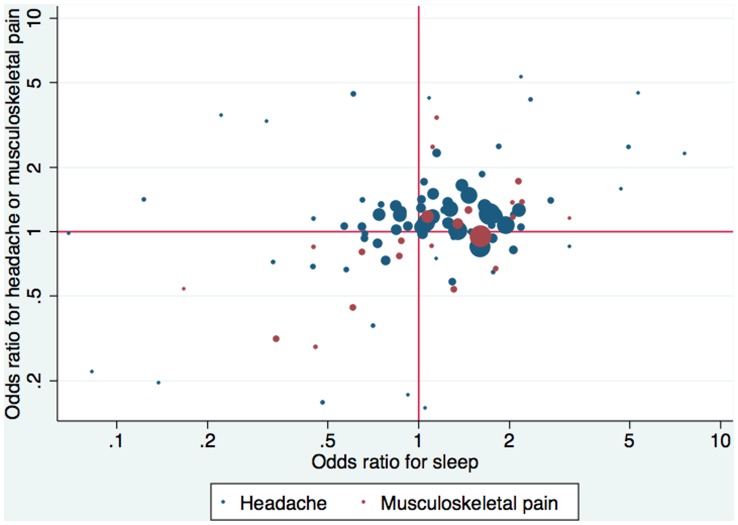
Correlation between treatment effect size (odds ratio) for sleep vs. headache or musculoskeletal pain. Not shown are 6 (3 on each type of pain) comparisons that have effects outside the range.

For headache and musculoskeletal pain, 69 interventions showed concordant direction of effect estimates between pain and sleep disturbance outcomes and 36 showed discordant direction (*p*<0.0001). As shown in Figure 1 and in Table 2, there was some modest correlation in the effect sizes for headache versus sleep disturbance in both weighted and unweighted analyses, and in weighted analyses for musculoskeletal pain versus sleep disturbance, but this was not nominally statistically significant. When both headache and musculoskeletal pain were considered together against sleep, weighted correlation was 0.17 (*p* = 0.092).

**Figure 2:**
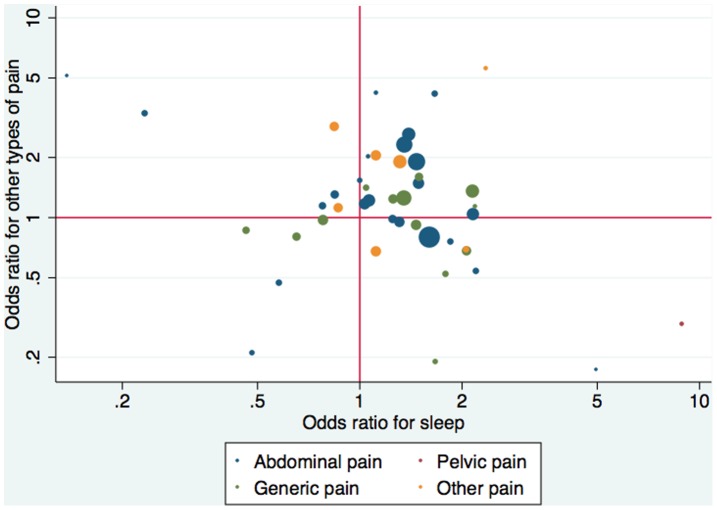
Correlation between treatment effect size (odds ratio) for sleep vs. any other type of pain outcome. Not shown are 2 comparisons that have effects outside the range (1 for abdominal pain and 1 for pelvic pain).

For the other 4 pain types there were 27 interventions with concordant direction of effect estimates and 19 with discordant direction (*p* = 0.095). As shown in Figure 2 and Table 2, there was no evidence for correlation between effect sizes.

**Table 2:** Correlations of the effect sizes for sleep versus pain outcomes.

	Unweighted analyses		Weighted analyses	
Type of pain	Correlation coefficient	*p*-value	Correlation coefficient	*p*-value
Headache	0.28	0.013	0.11	0.35
Musculoskeletal pain	−0.03	0.89	0.32	0.12
Abdominal pain	−0.13	0.55	−0.08	0.72
Pelvic pain	NP	NP	NP	NP
Generic pain	−0.09	0.77	0.10	0.74
Other type of pain	0.19	0.69	−0.06	0.9
Headache & musculoskeletal pain	0.14	0.15	0.17	0.092
All other 4 types of pain	0.06	0.69	−0.08	0.62

NP, not pertinent, because only two comparisons were available.

### Primary and Non-primary Outcomes

There were very limited data where both pain and sleep were primary outcomes (n = 5 comparisons), and sparse data where at least one of the two was a primary outcome (n = 17 comparisons) to allow any conclusive inferences (Table 3). However, even when both pain and sleep disturbance were non-primary outcomes, it was more likely for the direction of the effect estimates to be in concordant vs. discordant direction for sleep and headache (49 vs. 30, *p* = 0.003) and musculoskeletal pain (16 vs. 4, *p* = 0.047), but not for the other types of pain. Treatment effect sizes did not seem to correlate for pain and sleep.

**Table 3:** Analyses for primary vs. non-primary outcome.

Sleep and pain outcomes	Type of pain	Concordant direction of effects	Discordant direction of effects	*p*-value for binomialtest	Weighted correlation coefficient	*p*-value for correlation
Both non-primary outcomes	Headache	49	30	0.003	0.09	0.43
	Musculoskeletal pain	16	4	0.047	0.25	0.28
	Abdominal pain	14	10	0.25	−0.08	0.72
	Generic pain	9	4	0.05	0.10	0.74
	Other type of pain	3	4	0.59	−0.06	0.90
One or both of them primary outcomes	Headache	4	3	0.59	0.07	0.88
	Musculoskeletal pain	5	2	0.11	0.03	0.95
	Abdominal	0	1	NA	NA	NA
	Pelvic	1	1	NA	NA	NA

NA, not applicable.

### Other Sensitivity and Subgroup Analyses

Among trials with data on both pain and sleep, there were 90 comparisons with concordant direction of effects and 48 with opposite direction of effects (*p*<0.0001). The preponderance of concordant results was observed for headache (48 vs. 27, *p* = 0.0006) and musculoskeletal pain (15 vs. 6, *p* = 0.006), but also for others types of pain (27 vs. 15, *p* = 0.01). Among double-blinded trials, there were 79 comparisons with concordant direction of effects and 46 with discordant direction of effects (*p*<0.0001). The preponderance of concordant results was driven by headache (43 vs. 26, *p* = 0.004) and musculoskeletal pain (13 vs. 3, *p* = 0.0004), and not by others types of pain (23 vs. 17, *p* = 0.18). Finally, among trials that had compared an active intervention against an inactive control (placebo/no treatment) there were 58 comparisons with concordant direction of effects and 29 with discordant direction of effect (*p*<0.0001).

## Discussion

Across 151 treatment comparisons with pain and sleep outcomes, it was almost twice more common to see concordance in the direction of the treatment effects between sleep and pain rather than point estimates in opposite directions. Sleep outcomes were mostly concordant with headache and musculoskeletal pain. In the evaluated topics where most evidence was available, drug treatments that worsened sleep almost always also tended to worsen headache or musculoskeletal pain. Correlations in the magnitude of the effect size for sleep and pain were weak, but the strongest hints were again seen for headache and musculoskeletal pain. The concordant pattern was seen even though in most trials sleep and pain were not primary outcomes. The same picture emerged when we limited analyses to trials that provided data on both sleep and pain, double-blinded trials and trials with inactive controls.

Current evidence suggests a bidirectional relationship between sleep and pain where pain results in sleep disturbance and disturbed sleep enhances pain [17], [20]–[22], [26] with 50–89% of chronic pain patients also experiencing concomitant sleep problems [42]. Several chronic disease states including headache [43], and musculoskeletal syndromes (back pain [8], [10], fibromyalgia [11], [24], [25], [44], and temporomandibular joint disorder [15]) manifest both pain and sleep disturbance as major symptoms [16], [45]. Furthermore, poor sleep in fibromyalgia patients could predict pain at 1 year follow up [24], while longitudinal evidence from 12,350 healthy women has shown that sleep problems increased the risk of fibromyalgia in a dose–dependent manner [25]. The presence of restorative sleep in patients suffering from fibromyalgia predicted the resolution of pain at 15 months [44].

We found substantial evidence to support that sleep and pain outcomes are concordant predominantly when headache or musculoskeletal pain were involved. In the typical situation we encountered, drugs with poor sleep outcomes also had more headache or musculoskeletal pain. These findings confirm our hypothesis and prior evidence from both the general population [6] and chronic pain patients in specific [9], [16], [46]. Insomnia is a common symptom in patients with headache [43] and information on sleep disturbances is crucial in the clinical investigation of these patients [28]. Similarly, musculoskeletal pain is the most frequent complain for sleep disturbance in the general population [6], and, these two are major manifestations of common pain disorders like chronic back pain, fibromyalgia, and osteoarthritis [16].

The evidence for concordance of sleep with other types of pain outcomes was weak. For some cases where both sleep and abdominal pain worsened significantly, this could be attributed to specific side effects of the corresponding drugs. Sleep disturbance, gastrointestinal upset, stomach pain/cramps, and headaches are known side-effects of cholinesterase inhibitors, naltrexone [47], and sertraline, whereas goserelin can also cause sleep disturbance [48]. The choice of comparator may affect sometimes the concordance of sleep and pain outcomes. For example, goserelin was worse than oral contraceptives in both sleep outcome and pain control for endometriosis [38] but it was worse for sleep and better for pain when compared with placebo [40]. While we found many examples, where medical treatments worsened both pain and sleep, we found sparse conclusive data on medical treatments that can improve both of these outcomes. The observed beneficial effect of ozzlo pillow on both sleep and back pain is probably not surprising given the strong bidirectional relationship of the two outcomes [49].

Additionally, we identified nominally significant effects in the opposite direction for sleep disturbance and generic pain and abdominal pain with sertraline and atovaquone-proguanil respectively. For the case of sertraline, disturbed sleep has been identified as a common side effect of all SSRIs, whereas pain may be a common symptom for the conditions that it is indicated for. On the contrary, atovaquone-proguanil is known to cause abdominal pain, while amodiaquine, the active comparator, demonstrates insomnia as one of its main toxicities [48].

We should acknowledge some limitations in our work. First, although we accumulated data from 385 trials, most of them were small, and thus many of the effect estimates carried large uncertainty. This introduces noise in the effect size and may also affect the direction of the estimate. Noise would weaken, if anything, the observed concordance of outcomes and correlation of effect sizes. Second, both pain and sleep are influenced by a multitude of other factors and many of these are intervention-specific or disease-specific. However, overarching patterns emerge when many conditions and treatments are examined. Third, we limited our analyses to binary outcomes for consistency, but a perusal of the Cochrane database suggested that very few topics would have been added, if we had included also continuous outcomes. Fourth, we cannot exclude the possibility of selective reporting for some outcomes in several trials. For example, perhaps some outcomes were more likely to be reported, if they had statistically significant results. Although many trials did not report both sleep and pain outcomes, a sensitivity analysis limited to trials reporting both yielded similar inferences. Fifth, each of the pain categories included different types of pain that may be quite different and may not have the same exact relationship with how they might affect sleep outcomes. Moreover, some types of pain syndromes were not represented in our data, e.g. fibromyalgia.

Acknowledging these caveats, this empirical assessment identifies a common thread with concordance between headache and musculoskeletal types of pain and sleep outcomes. The concordance is not absolute, there are exceptions to this pattern, and it is not possible to predict reliably the exact magnitude of the effect of an intervention on pain based on the magnitude of its effect on sleep and vice versa for most medical interventions. However, it seems that several drugs may cause both sleep and pain problems. Many trials and systematic reviews currently address only one of these two outcomes. More routine assessment of both of these types of outcomes may need to be integrated in evaluating the response of patients to diverse treatments, since these adverse effects may coexist. Conversely, more prospective clinical trials are needed to identify the extent to which treatments that provide effective pain relief can also enhance sleep and whether patients with chronic pain disorders could potentially benefit from interventions at the level of sleep.

## Supporting Information

Figure S1 **Flow chart for the selection of eligible reviews.**
(DOC)

Table S1 **Eligible sleep-related outcomes which were all considered together under the inclusive term sleep disturbance.**
(DOC)

Table S2 **Eligible pain-related outcomes.**
(DOC)

Table S3 **The eligible comparisons on sleep and pain.** Trials have been synthesized under fixed-effects.
(DOC)
