# Supplementary figures and images for: Concordance of Sleep and Pain Outcomes of Diverse Interventions: An Umbrella Review
Source: PLoS One. 2012 Jul 17;7(7):e40891. doi: 10.1371/journal.pone.0040891 (PMC3398909; doi:10.1371/journal.pone.0040891)

**Figure S1. Flow chart for the selection of eligible reviews**


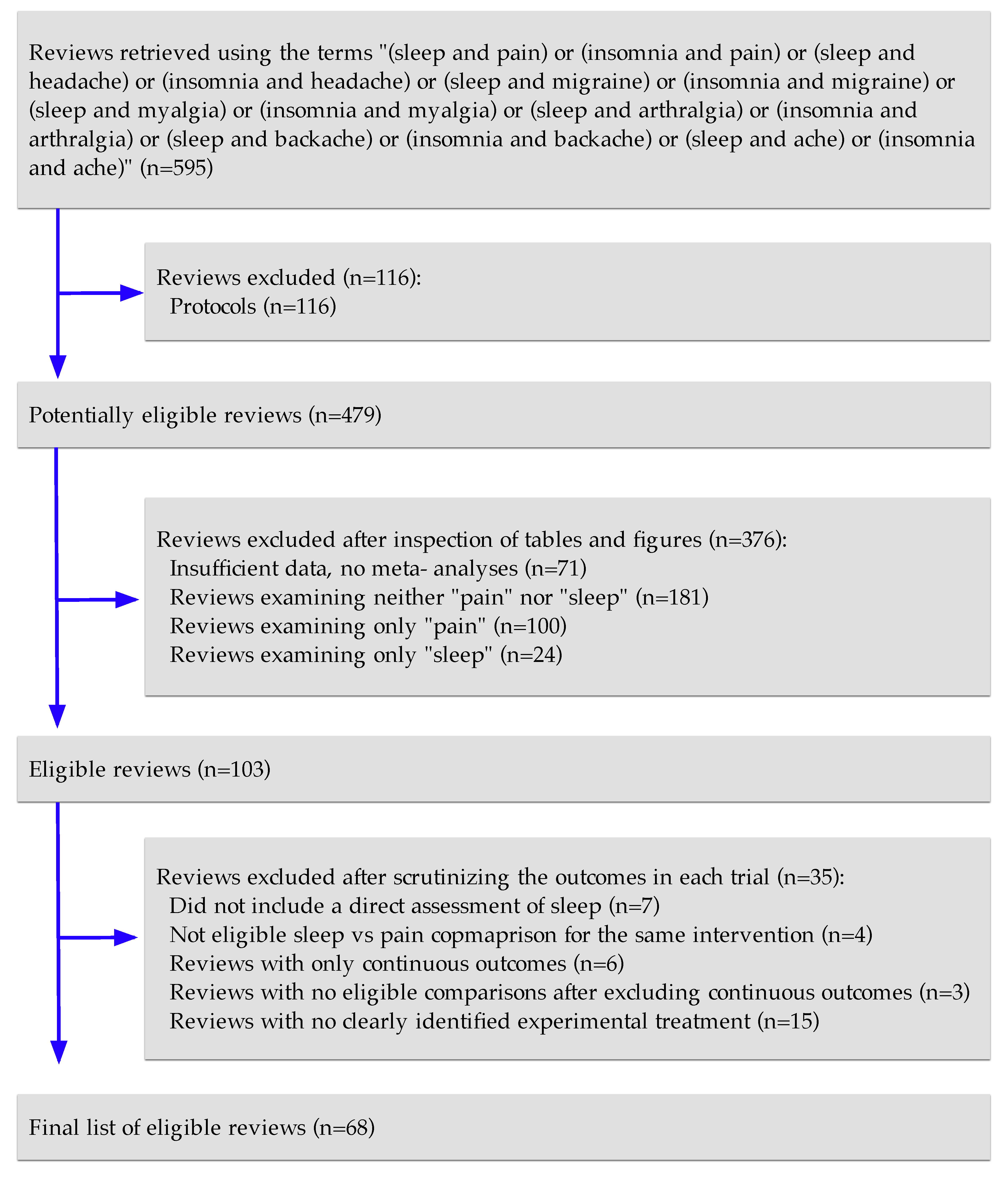

Supplement: Figure S1 — Flow chart for the selection of eligible reviews. (DOC) [file pone.0040891.s001.doc]
