# Supplementary material for: Concordance of Sleep and Pain Outcomes of Diverse Interventions: An Umbrella Review
Source: PLoS One. 2012 Jul 17;7(7):e40891. doi: 10.1371/journal.pone.0040891 (PMC3398909; doi:10.1371/journal.pone.0040891)
Supplement: Table S1 — Eligible sleep-related outcomes which were all considered together under the inclusive term sleep disturbance. (DOC) [file pone.0040891.s002.doc]

**Table S1. Eligible sleep-related outcomes which were all considered together under the inclusive term *sleep disturbance*.**

| **Sleep-related outcomes** | **Specific sleep-related assessments** |
| --- | --- |
| **Insomnia / sleep disturbance** | Difficulty falling asleep  Disturbed sleep  Early awakening  Functional outcomes of sleep questionnaire  Insomnia (sleep onset latency in min)  Insomnia/sleep disturbance  Insomnia  Improvement in sleep quality  Improvement in sleep disturbance  Inability to sleep  Insomnia or sleeplessness  No improvement in sleep  Nights without sleep disturbance (change in %)  Post-treatment sleep onset latency (min)  Post-treatment sleep quality score  Stimulation / insomnia  Sleep quality score (change in %)  Sleep benefit (moderate or better)  Self-rated improvement of insomnia  Self-rated improvement of insomnia ≥ 50%  Sleep disorders  Sleep disturbance  Sleep disturbance score  Sleep problems  Sleep onset difficulties  Sleep quality (Pittsburgh sleep quality index)  Sleep quality not good  Sleep quality OK at most |
| **Sleep disruption** | Arousal index  Arousals  Awakening from sleep  Decreased sleep duration or quality  Early awakening  Nocturnal awakening  Post-treatment mid-sleep awakenings  Post-treatment sleep efficiency  Restless sleep by questionnaire  Sleep interruption  Shortened sleep  Sleep disruption  Total sleep duration score (change) |
