# Supplementary material for: Concordance of Sleep and Pain Outcomes of Diverse Interventions: An Umbrella Review
Source: PLoS One. 2012 Jul 17;7(7):e40891. doi: 10.1371/journal.pone.0040891 (PMC3398909; doi:10.1371/journal.pone.0040891)
Supplement: Table S2 — Eligible pain-related outcomes. (DOC) [file pone.0040891.s003.doc]

**Table S2. Eligible pain-related outcomes.**

| **Pain-related outcomes** | **Specific pain-related assessments** |
| --- | --- |
| **Abdominal pain** | Abdominal pain at "x" weeks  Abdominal pain/distension/cramping  Abdominal pain  Abdominal pain at the end of titration  Abdominal pain before the end of treatment  Abdominal pain before the end of treatment by ≥ 6 mos  Abdominal pain  Abdominal pain - short term  Abdominal pain or discomfort  Stomach pain  Stomach ache |
| **Generic pain** | Generic pain |
| **Headache** | Headache at "x" years  Headache - short term  Headache - medium term  Headache and migraine  Headache at "x" weeks  Headache at "x" months  Headache at the end of titration  Headache before the end of treatment by ≥ 6 months  Headache before the end of treatment  Headache between "x" and "y" weeks  Headache ratings over 3.5 years follow-up  Migraine |
| **Musculoskeletal pain** | Change in pain from baseline  Pain at 1 year  Severe or moderate pain at "x" months  Arthtralgia  Arthralgia at 1 year  Arthralgia at 6 months  Arthralgia before the end of trt by ≥ 6 mos  Back pain  Back pain at "x" weeks  Back pain (change from baseline)  Back pain before the end of trt by ≥ 6 mos  Back or pelvic pain (during labour)  Backache  Backache improvement  Bodily pain score (SF36)  Jaw pain intensity upon awakening  Joint pain  Joint pain score  Joint pain score (change from baseline)  Muscle cramps/myalgia  Muscle pain severity on palpation  Muscular, limb pain  Myalgia  Muscle pain  Muscle pain severity  Musculoskeletal pain  Myalgia/fatigue  Pain in limb  Pain in muscle/joints score  Pain - body pain  Pain on movement  Pain in arms or legs/arthralgia  Temporomandibular joint pain |
| **Other pain** | Abdominal scar pain  Chest pain  Breast pain  Breast pain / tension  Breast tenderness  Injection pain  Pain at injection site  Toothache  Pharyngolaryngeal pain  Post-herpetic neuralgia  Postoperative pain  Procedural pain  Side pains |
| **Pelvic pain** | Dysmenorrhea  Dysmenorrhea at the end of treatment (reduction)  Dysmenorrhea - relief of painful symptoms  Dyspareunia  Dyspareunia - relief of painful symptoms  Menstrual pain  Menstrual pelvic pain  Non-menstrual pain at the end of treatment  Non-menstrual pain at "x" months  Pelvic tenderness  Pelvic tenderness - relief of painful symptoms  Pelvic pain - relief of painful symptoms |
