# Supplementary material for: Concordance of Sleep and Pain Outcomes of Diverse Interventions: An Umbrella Review
Source: PLoS One. 2012 Jul 17;7(7):e40891. doi: 10.1371/journal.pone.0040891 (PMC3398909; doi:10.1371/journal.pone.0040891)
Supplement: Table S3 — The eligible comparisons on sleep and pain. Trials have been synthesized under fixed-effects. (DOC) [file pone.0040891.s004.doc]

**Table S3. The eligible comparisons on sleep and pain.**

***Trials have been synthesized under fixed-effects.***

| **Review** | **Comparison** | **OR (95% CI) for sleep** | **OR (95% CI) for pain** | **Type of pain** |
| --- | --- | --- | --- | --- |
| CD000434 | Early mobilization (within or up to 1 week) versus immobilization for 3 weeks for treating proximal humeral fractures in adults | 0.46 (0.10-1.98) | 0.29 (0.09-0.92) | Musculoskeletal pain |
| CD000440 | Risperidone vs typical neuroleptic medication for schizophrenia | 1.05 (0.83-1.34) | 1.06 (0.84-1.36) | Headache |
| CD000547 | GnRH analogue treatment vs lynestrenol before hysterectomy or myomectomy for uterine fibroids | 2.20 (0.21-22.59) | 5.25 (1.04-26.55) | Headache |
| CD001019 | Goserelin vs oral contraceptives for pain associated with endometriosis | 19.00 (1.03-350.73) | 0.58 (0.15-2.35) | Headache |
|  |  |  | 3.00 (0.117-76.79) | Musculoskeletal pain |
|  |  |  | 20.33 ( 1.08-381.56) | Pelvic |
| CD001130 | SSRIs vs placebo for dysthymia | 1.55 (1.03-2.34) | 0.85 (0.60-1.19) | Headache |
|  | TCAs vs placebo for dysthymia | 0.70 (0.43-1.26) | 0.87 (0.58-1.32) | Headache |
|  | MAOIs vs placebo for dysthymia | 1.64 (0.75-3.57) | 1.85 (0.86-3.99) | Headache |
| CD001139 | Ozzlo pillow vs standard pillow for preventing and treating pelvic and back pain in pregnancy | 0.34 (0.19-0.62) | 0.31 (0.17-0.57) | Musculoskeletal pain |
| CD001162 | Sulpiride vs typical antipsychotics for schizophrenia | 1.03 (0.62-1.71) | 1.41 (0.85-2.34) | Headache |
| CD001190 | Donepezil vs placebo for dementia due to Alzheimer's disease | 2.16 (1.53-3.04) | 1.25 (0.97-1.60) | Headache |
|  |  |  | 1.70 (0.87-3.33) | Musculoskeletal pain |
|  |  |  | 1.03 (0.64-1.66) | Abdominal pain |
|  |  |  | 1.35 (0.90-2.03) | Generic pain |
| CD001191 | Rivastigmine 1-4 mg (total divided into two doses daily) vs placebo for Alzheimer's disease | 1.04 (0.64-1.68) | 0.97 (0.69-1.38) | Headache |
|  |  |  | 1.16 (0.72-1.88) | Abdominal pain |
|  | Rivastigmine 6-12 mg (total divided into two doses daily) vs placebo for Alzheimer's disease | 1.40 (0.93-2.09) | 1.64 (1.25-2.15) | Headache |
|  |  |  | 2.60 (1.77-3.82) | Abdominal pain |
| CD001385 | Long-acting beta2-agonists vs placebo for chronic asthma in adults and children where background therapy contains varied or no inhaled corticosteroid | 2.06 (0.61-6.92) | 1.19 (0.99-1.43) | Headache |
|  |  |  | 1.37 (0.63-2.97) | Musculoskeletal pain |
|  |  |  | 0.69 (0.11-4.17) | Other pain |
| CD001396 | SSRI versus placebo for premenstrual syndrome | 1.80 (1.32-2.47) | 1.18 (0.92-1.51) | Headache |
|  |  |  | 0.52 (0.17-1.60) | Generic pain |
|  |  |  | 0.67 (0.22-1.99) | Musculoskeletal pain |
| CD001715 | Sertindole versus haloperidol for schizophrenia | 0.88 (0.53-1.45) | 1.24 (0.91-1.67) | Headache |
|  |  |  | 0.90 (0.47-1.71) | Musculoskeletal pain |
|  | Sertindole versus placebo for schizophrenia | 0.65 (0.37-1.14) | 1.05 (0.63-1.74) | Headache |
|  |  |  | 0.80 (0.38-1.68) | Musculoskeletal pain |
| CD001765 | SSRIs versus placebo for obsessive compulsive disorder | 1.96 (1.57-2.45) | 1.06 (0.85-1.33) | Headache |
| CD001867 | Naltrexone vs placebo for alcohol dependence | 1.36 (1.04-1.77) | 1.01 (0.85-1.19) | Headache |
|  |  |  | 1.25 (0.89-1.76) | Generic pain |
|  |  |  | 2.30 (1.61-3.27) | Abdominal pain |
|  |  |  | 1.08 (0.76-1.52) | Musculoskeletal pain |
| CD001943 | Loxapine vs placebo for schizophrenia | 0.48 (0.15-1.52) | 0.16 (0.04-0.58) | Headache |
|  |  |  | 0.21 (0.06-0.77) | Abdominal pain |
|  | Loxapine vs typical antipsychotics for schizophrenia | 1.00 (0.44-2.27) | 1.52 (0.31-7.53) | Abdominal pain |
| CD001944 | Thioridazine vs placebo for schizophrenia | 0.45 (0.11-1.90) | 1.14 (0.41-3.13) | Headache |
| CD001945 | Ziprasidone vs typical antipsychotics for schizophrenia and severe mental illness | 1.29 (0.70-2.40) | 0.58 (0.27-1.27) | Headache |
|  | Ziprasidone vs placebo for schizophrenia and severe mental illness | 0.78 (0.43-1.43) | 0.73 (0.50-1.07) | Headache |
|  |  |  | 0.97 (0.65-1.43) | Generic pain |
|  |  |  | 1.14 (0.42-3.10) | Abdominal pain |
| CD001948 | Zotepine vs placebo for schizophrenia | 0.47 (0.21-1.01) | 0.86 (0.40-1.84) | Generic pain |
|  | Zotepine vs typical antipsychotics for schizophrenia | 0.66 (0.42-1.02) | 1.40 (0.41-4.77) | Headache |
|  |  |  | 0.80 (0.37-1.71) | Generic pain |
| CD001949 | Pimozide vs placebo for schizophrenia or related psychoses | 0.22 (0.02-2.28) | 3.50 (0.64-19.20) | Headache |
| CD002304 | Clotiapine versus standard medication - other antipsychotics for acute psychotic illnesses | 2.36 (0.36-15.46) | 4.13 (0.88-19.27) | Headache |
|  |  |  | 5.51 (0.25-122.09) | Other pain |
| CD002317 | Tricyclic drugs vs placebo for depression in children and adolescents | 1.87 (0.88-3.94) | 1.15 (0.68-1.95) | Headache |
| CD002745 | Amantadine vs placebo for influenza A in children | 0.45 (0.12-1.75) | 0.68 (0.45-1.04) | Headache |
|  |  |  | 0.84 (0.43-1.64) | Musculoskeletal pain |
|  | Rimantadine vs placebo for influenza A in the elderly | 1.31 (0.48-3.59) | 0.93 (0.36-2.42) | Headache |
| CD002778 | Stabilisation splint vs minimal/no treatment for temporomandibular pain dysfunction syndrome | 1.11 (0.31-4.04) | 0.86 (0.23-3.25) | Musculoskeletal pain |
| CD002869 | Group maternal education versus routine care for childbirth or parenthood or both | 0.61 (0.35-1.07) | 4.41 (1.20-16.14) | Headache |
|  |  |  | 0.44 (0.25-0.78) | Musculoskeletal pain |
| CD002885 | Xanthine versus beta2-agonists for asthma in children | 1.86 (0.62-5.61) | 2.50 (1.01-6.20) | Headache |
|  |  |  | 0.75 (0.26-2.15) | Abdominal pain |
| CD002904 | Glucocorticosteroids plus interferon vs interferon plus no intervention/placebo for viral hepatitis C | 3.16 (0.12-82.64) | 0.85 (0.17-4.20) | Headache |
|  |  |  | 1.15 (0.24-5.39) | Musculoskeletal pain |
| CD003082 | Haloperidol versus placebo for schizophrenia | 1.76 (0.93-3.33) | 1.07 (0.58-1.98) | Headache |
| CD003154 | Memantine vs placebo for mild to severe dementia | 0.93 (0.59-1.48) | 1.05 (0.67-1.63) | Headache |
| CD003443 | Perphenazine vs placebo for schizophrenia | 0.33 (0.11-1.03) | 0.71 (0.14-3.60) | Headache |
| CD003794 | Fluticasone/salmeterol (FPS) versus placebo for chronic obstructive pulmonary disease | 0.07 (0.01-1.36) | 0.97 (0.74-1.27) | Headache |
| CD003902 | Theophylline vs placebo for chronic obstructive pulmonary disease | 0.31 (0.01-8.27) | 3.26 (0.12-88.35) | Headache |
| CD004043 | Risperidone vs placebo for acute mania | 0.57 (0.27-1.20) | 1.05 (0.67-1.66) | Headache |
| CD004050 | Bright light vs inactive placebo for non-seasonal depression | 5.00 (1.12-22.25) | 2.47 (0.76-8.05) | Headache |
|  |  |  | 0.17 (0.01-3.94) | Abdominal pain |
| CD004161 | Risperidone depot vs placebo for schizophrenia | 1.05 (0.55-2.01) | 1.70 (0.87-3.32) | Headache |
|  |  |  | 1.40 (0.46-4.27) | Generic pain |
| CD004258 | Pulsed electromagnetic field vs placebo for shoulder pain | 5.35 (0.25-116.32) | 4.46 (0.47-42.52) | Headache |
|  |  |  | 0.01 (0.001-0.17) | Musculoskeletal pain |
| CD004290 | TOR-I versus antimetabolites for primary immunosuppression in kidney transplant recipients | 1.15 (0.69-1.93) | 2.31 (1.32-4.03) | Headache |
|  |  |  | 3.41 (0.64-18.25) | Musculoskeletal pain |
| CD004332 | Acamprosate vs naltrexone for alcohol dependence | 0.71 (0.15-3.32) | 0.36 (0.07-1.95) | Headache |
|  | Acamprosate vs placebo for alcohol dependence | 1.31 (0.88-1.96) | 0.99 (0.70-1.42) | Headache |
|  |  |  | 0.95 (0.48-1.86) | Abdominal pain |
|  |  |  | 0.53 (0.28-1.02) | Musculoskeletal pain |
|  |  |  | 1.90 (0.83-4.37) | Other pain |
| CD004362 | Haloperidol vs placebo for acute mania | 0.67 (0.32-1.41) | 0.96 (0.45-2.09) | Headache |
| CD004395 | Donepezil vs placebo for vascular cognitive impairment | 2.07 (1.20-3.59) | 0.81 (0.48-1.37) | Headache |
|  |  |  | 0.68 (0.41-1.12) | Generic pain |
|  |  |  | 1.16 (0.52-2.62) | Musculoskeletal pain |
| CD004410 | New generation antipsychotics vs conventional antipsychotics (haloperidol) for first episode schizophrenia | 0.75 (0.36-1.59) | 1.33 (0.53-3.37) | Headache |
| CD004435 | Oral appliance versus continuous positive airways pressure for obstructive sleep apnoea | 0.67 (0.27-1.62) | 80.26 (10.36-622.11) | Musculoskeletal pain |
| CD004529 | Atovaquone-proguanil vs amodiaquine for treating uncomplicated malaria | 0.23 (0.08-0.68) | 3.31 (1.11-9.85) | Abdominal pain |
|  | Atovaquone-proguanil vs chloroquine for treating uncomplicated malaria | 1.06 (0.15-7.34) | 0.15 (0.02-0.90) | Headache |
|  |  |  | 2.00 (0.33-12.18) | Abdominal pain |
|  | Atovaquone-proguanil vs halofantrine for treating uncomplicated malaria | 0.58 (0.19-1.81) | 0.66 (0.28-1.54) | Headache |
|  |  |  | 0.47 (0.20-1.08) | Abdominal pain |
|  | Atovaquone-proguanil vs mefloquine for treating uncomplicated malaria | 0.14 (0.01-2.71) | 0.20 (0.01-4.13) | Headache |
|  |  |  | 5.11 (0.24-107.98) | Abdominal pain |
|  |  |  | 0.77 (0.42-1.40) | Abdominal pain |
| CD004578 | Aripiprazole vs placebo for schizophrenia | 1.05 (0.76-1.46) | 1.12 (0.77-1.62) | Headache |
|  | Aripiprazole vs typical antipsychotics for schizophrenia | 1.76 (1.16-2.70) | 0.93 (0.56-1.53) | Headache |
| CD005148 | Anti-obesity drugs +/- life style management vs placebo to reduce weight gain in schizophrenia | 1.78 (0.75-4.22) | 0.64 (0.14-2.92) | Headache |
|  | Antiparkinsonian drugs vs placebo to reduce weight gain in schizophrenia | 4.22 (1.29-13.78) | 15.62 (0.86-283.63) | Abdominal pain |
| CD005445 | Ribavirin/Interferon vs interferon for chronic hepatitis C | 1.61 (1.37-1.90) | 0.84 (0.69-1.04) | Headache |
|  |  |  | 0.79 (0.59-1.06) | Abdominal pain |
|  |  |  | 0.95 (0.85-1.06) | Musculoskeletal pain |
| CD005472 | Accupressure vs sham for insomnia | 0.17 (0.02-1.55) | 0.54 (0.17-1.71) | Musculoskeletal pain |
| CD005520 | Oral appliance vs no treatment for obstructive sleep apnoea in children | 0.08 (0.01-0.64) | 0.22 (0.03-1.36) | Headache |
| CD005593 | Cholinesterase inhibitor (optimum dose) vs placebo for Alzheimer's disease | 1.47 (1.10-1.97) | 1.47 (1.19-1.82) | Headache |
|  |  |  | 0.91 (0.47-1.78) | Generic pain |
|  |  |  | 1.90 (1.40-2.58) | Abdominal pain |
|  |  |  | 1.25 (0.69-2.25) | Musculoskeletal pain |
| CD005653 | Olanzapine vs placebo for borderline personality disorder | 0.67 (0.34-1.32) | 0.92 (0.57-1.50) | Headache |
| CD006103 | Varenicline (1.0mg 2/d) vs placebo for smoking cessation | 1.73 (1.44-2.08) | 1.20 (0.99-1.45) | Headache |
| CD006114 | Fluvoxamine vs TCAs for depression | 1.26 (0.82-1.92) | 1.08 (0.79-1.48) | Headache |
| CD006115 | Azapirone vs benzodiazepine for generalized anxiety disorder | 2.76 (0.91-8.32) | 1.40 (0.85-2.30) | Headache |
|  | Azapirones vs placebo for generalized anxiety disorder | 2.20 (1.04-4.67) | 1.04 (0.69-1.58) | Headache |
|  |  |  | 0.54 (0.16-1.84) | Abdominal pain |
|  |  |  | 1.13 (0.17-7.47) | Generic pain |
|  |  |  | 1.37 (0.52-3.64) | Musculoskeletal pain |
|  | Buspirone vs benzodiazepine for generalized anxiety disorder | 0.12 (0.03-0.60) | 1.40 (0.71-2.76) | Headache |
|  | Buspirone vs placebo for generalized anxiety disorder | 1.53 (0.55-4.24) | 0.92 (0.52-1.61) | Headache |
| CD006117 | Sertraline versus TCAs for depression | 1.67 (1.21-2.31) | 1.31 (0.99-1.74) | Headache |
|  |  |  | 4.13 (1.12-15.25) | Abdominal pain |
|  |  |  | 0.19 (0.04-0.99) | Generic pain |
| CD006332 | Alvimopan 12mg vs placebo for opioid-induced bowel dysfunction | 0.85 (0.57-1.27) | 1.01 (0.67-1.50) | Headache |
|  | Alvimopan 6mg vs placebo for opioid-induced bowel dysfunction | 1.03 (0.64-1.64) | 1.28 (0.80-2.04) | Headache |
| CD006369 | Paliperidone - any dose or flexible doses vs olanzapine (fixed dose 10 mg/day all short term) for schizophrenia | 1.12 (0.77-1.62) | 1.49 (1.00-2.21) | Headache |
|  |  |  | 0.67 (0.21-2.16) | Other pain |
|  |  |  | 2.46 (0.70-8.70) | Musculoskeletal pain |
|  | Paliperidone - any dose or flexible doses vs placebo (all short term) for schizophrenia | 0.87 (0.64-1.17) | 1.19 (0.85-1.66) | Headache |
|  |  |  | 1.11 (0.28-4.37) | Other pain |
|  |  |  | 0.77 (0.32-1.86) | Musculoskeletal pain |
|  | Paliperidone - any dose or flexible doses vs quetiapine (flexible dose all short term) for schizophrenia | 1.22 (0.60-2.47) | 1.26 (0.65-2.41) | Headache |
| CD006391 | Benzodiazepines as sole treatment vs placebo as sole treatment for schizophrenia | 1.15 (0.05-28.92) | 0.75 (0.18-3.02) | Headache |
|  | Benzodiazepines vs antipsychotics for schizophrenia | 0.93 (0.02-48.49) | 0.17 (0.01-3.74) | Headache |
| CD006564 | Dopamin agonists vs placebo/L-dopa in early Parkinson's disease | 1.28 (1.00-1.62) | 1.27 (0.95-1.68) | Headache |
| CD006617 | Aripiprazole vs typical antipsychotic drugs (short term /=12 weeks) for schizophrenia | 0.85 (0.60-1.20) | 1.31 (0.91-1.89) | Headache |
|  |  |  | 1.30 (0.49-3.45) | Abdominal pain |
|  |  |  | 2.85 (0.69-11.77) | Other pain |
| CD006622 | Aripiprazole vs placebo for schizophrenia | 1.12 (0.84-1.50) | 1.16 (0.88-1.54) | Headache |
|  |  |  | 4.21 (0.23-75.42) | Abdominal pain |
|  |  |  | 2.02 (0.63-6.44) | Other pain |
| CD006722 | Psychostimulants vs placebo as adjunct to antidepressant treatment for depression | 1.24 (0.62-2.50) | 1.10 (0.65-1.85) | Headache |
| CD006815 | Hydroxyzine vs placebo for generalized anxiety disorder | 1.09 (0.32-3.73) | 4.21 (0.46-38.49) | Headache |
| CD006918 | Risperidone vs placebo for schizophrenia | 1.03 (0.75-1.41) | 1.04 (0.75-1.44) | Headache |
| CD007166 | COMTI vs placebo as adjuvant treatment to levodopa therapy in Parkinson’s disease patients with motor complications | 1.50 (0.99-2.26) | 0.99 (0.31-3.14) | Headache |
|  |  |  | 1.48 (0.88-2.48) | Abdominal pain |
|  |  |  | 1.58 (0.70-3.60) | Generic pain |
|  | Dopamine agonists vs placebo as adjuvant treatment to levodopa therapy in Parkinson’s disease patients with motor complications | 1.26 (0.93-1.69) | 1.36 (0.83-2.22) | Headache |
|  |  |  | 0.98 (0.38-2.52) | Abdominal pain |
|  |  |  | 1.23 (0.63-2.42) | Generic pain |
| CD007503 | Antidepressants for depression in physically ill people | 0.75 (0.50-1.11) | 1.20 (0.89-1.62) | Headache |
| CD007621 | Natalizumab + IFN vs IFN for relapsing remitting multiple sclerosis | 1.07 (0.79-1.45) | 1.09 (0.86-1.37) | Headache |
|  |  |  | 1.20 (0.73-2.00) | Abdominal pain |
|  |  |  | 1.17 (0.94-1.46) | Musculoskeletal pain |
| CD007779 | Atypical antipsychotics vs levomepromazine vs for schizophrenia | 4.70 (0.48-46.23) | 1.58 (0.24-10.61) | Headache |
| CD008373 | Antidepressants vs placebo for opioid dependence | 7.63 (0.38-151.87) | 2.30 (0.87-6.11) | Headache |
| CD008475 | GnRHas vs placebo for pain associated with endometriosis | 8.89 (2.31-34.25) | 0.29 (0.13-0.68) | Pelvic |
